# IGF-1 induces sex-specific oxidative damage and mortality in a songbird

**DOI:** 10.1007/s00442-024-05587-y

**Published:** 2024-07-16

**Authors:** Ádám Z. Lendvai, Zsófia Tóth, Katharina Mahr, Janka Pénzes, Sarah Vogel-Kindgen, Bruno A. Gander, Csongor I. Vágási

**Affiliations:** 1 Department of Evolutionary Zoology and Human Biology, University of Debrecen, Debrecen, Hungary; 2 Pál Juhász-Nagy Doctoral School of Biology Environmental Sciences, University of Debrecen, Debrecen, Hungary; 3 Department of Interdisciplinary Life Sciences, University of Veterinary Medicine, Konrad Lorenz Institute of Ethology, Vienna, Austria; 4 Evolutionary Ecology Group, Hungarian Department of Biology and Ecology, Babeş-Bolyai University, Cluj-Napoca, Romania; 5 Institute of Pharmaceutical Sciences, ETH Zurich, Zurich, Switzerland; 6 Department of Biology, Lund University, Lund, Sweden

**Keywords:** IGF-1, Oxidative stress, Lifespan, Fitness, Somatotropic axis

## Abstract

The insulin-like growth factor 1 (IGF-1) is a pleiotropic hormone that regulates essential life-history traits and is known for its major contribution to determining individual ageing processes. High levels of IGF-1 have been linked to increased mortality and are hypothesised to cause oxidative stress. This effect has been observed in laboratory animals, but whether it pertains to wild vertebrates has not been tested. This is surprising because studying the mechanisms that shape individual differences in lifespan is important to understanding mortality patterns in populations of free-living animals. We tested this hypothesis under semi-natural conditions by simulating elevated IGF-1 levels in captive bearded reedlings, a songbird species with an exceptionally fast pace of life. We subcutaneously injected slow-release biodegradable microspheres loaded with IGF-1 and achieved a systemic 3.7-fold increase of the hormone within the natural range for at least 24 h. Oxidative damage to lipids showed marked sexual differences: it significantly increased the day after the manipulation in treated males and returned to baseline levels four days post-treatment, while no treatment effect was apparent in females. Although there was no overall difference in survival between the treatment groups, high initial (pre-treatment) IGF-1 and low post-treatment plasma malondialdehyde levels were associated with enhanced survival prospects in males. These results suggest that males may be more susceptible to IGF-1-induced oxidative stress than females and quickly restoring oxidative balance may be related to fitness. IGF-1 levels evolve under opposing selection forces, and natural variation in this hormone’s level may reflect the outcome of individual optimization.

## Introduction

Mortality is a pivotal demographic parameter that profoundly influences fitness and is central to our understanding of the patterns contributing to individual differences in longevity. Within the realm of genetic and physiological mechanisms shaping animal longevity, the insulin/insulin-like growth factor 1 signalling (IIS) pathway stands out as a key regulator. This evolutionarily conserved pathway, present throughout the animal kingdom, has demonstrated associations with longevity in diverse species, including worms, insects and vertebrates (Bartke 2017). The primary ligand of the IIS pathway in vertebrates, the peptide hormone insulin-like growth factor 1 (IGF-1) has an antagonistic pleiotropic effect on different fitness components: it stimulates growth and reproduction but increases mortality (Dantzer and Swanson 2012). Conversely, a repressed activity of the IIS pathway augments self-maintenance or survival functions, resulting in an extended lifespan (Kenyon 2010; Lind et al. 2019).

Despite the robust patterns observed, the direct mechanisms by which a repressed IIS activity extends lifespan are not fully understood. The prevailing hypothesis is that the positive effects of blunted IIS signalling are partly due to increased resistance to oxidative stress (Holzenberger et al. 2003; reviewed by Tatar et al. 2003; Kenyon 2010; Dantzer and Swanson 2012). However, the relationship between IGF-1 and oxidative status is somewhat paradoxical. On the one hand, IGF-1 activates enzymatic antioxidant defences (most notably, glutathione peroxidase) and therefore is considered to have protective roles against oxidative stress, at least in rodents (Sukhanov et al. 2007; Higashi et al. 2010; Aksu et al. 2013; Ayadi et al. 2016; Montivero et al. 2021; Arjunan et al. 2023). On the other hand, IGF-1 is intricately linked with cellular metabolism and growth and may cause increased production of reactive oxygen species (Papaconstantinou 2009).

This disparity may, in part, be attributable to the fact that resistance to oxidative stress may not be equally experienced by males and females. In fact, sex-specific effects of IGF-1 regarding its protective or adverse roles have been documented in laboratory model organisms. For instance, improved stress resistance and longevity due to reduced IGF-1 signalling are much more profound in female mice than male mice (Holzenberger et al. 2003). High serum IGF-1 levels also induce bone loss in female mice only, while in males the effect is the opposite (Elis et al. 2011). Sex-specific effects of IGF-1 may also be observed in the regulation of the immune response (Pinto-Benito et al. 2022), which may contribute to a general sex difference in immunity, with potential implications for divergent longevities (May 2007; Vincze et al. 2022). Despite its importance, the underlying mechanisms of sexual differences in physiology and their repercussions for mortality patterns remain often unknown and warrant further research.

However, the role of IGF-1 in coordinating fitness and oxidative stress has almost exclusively been performed in the laboratory and studies in wild animals are surprisingly scarce (reviewed by Dantzer and Swanson 2012; Lodjak and Verhulst 2020). It is still contentious whether a high IGF-1 titre triggers oxidative damage, and this hypothesis has never been tested in any wild species (Dantzer and Swanson 2012; Lodjak and Verhulst 2020).

The aim of our study was to address this knowledge gap by exploring the consequences of short-term elevated IGF-1 levels on oxidative damage in male and female individuals of a wild bird species. We conducted an experiment with juvenile bearded reedlings (*Panurus biarmicus*), a common Eurasian passerine, previously used in several behavioural (e.g. Romero-Pujante et al. 2002; Hoi and Griggio 2012) and physiological studies, including that of IGF-1 (Tóth et al. 2018, 2022; Mahr et al. 2020; reviewed by Lendvai 2023). Plasma IGF-1 levels were experimentally increased by using a novel and minimally invasive manipulation technique that consisted of a single subcutaneous injection of IGF-1 encapsulated in slow-release biodegradable microspheres (treatment group) or dispersion medium without IGF-1 (control group) (Meinel et al. 2001; Mahr et al. 2023). To this end, we chose malondyaldehide (MDA) as a surrogate measure of oxidative damage due to its stability as a byproduct of lipid peroxidation, quantifiable nature allowing for comparisons across samples, and widespread use in scientific research. Malondialdehyde (MDA) is a carbonyl compound that results from the peroxidative degeneration of membrane lipids, and thus, it is a widely used marker of oxidative stress (Del Rio et al. 2005). MDA and other carbonyl compounds have longer half‐life than reactive oxygen species and can cross membranes to cause damage to cell macromolecules distant to their place of formation (e.g. Monaghan et al. 2009; Pamplona and Barja 2011; Sohal and Orr 2012). MDA has been shown to scale inversely with maximum lifespan in a comparison across birds (Vágási et al. 2019). Considering the unknown significance of even a temporary upsurge of oxidative damage, we also monitored long-term fitness consequences by recording the mortality of individual reedlings in captivity over 16 months after the start of the experiment.

## Material and methods

### Study species, experimental setup, mortality

The bearded reedlings (16 females and 25 males) were caught with mist nets at Hortobágy-Halastó (N47.6211, E21.0757) between July 28 and 30, 2017. Only juveniles (i.e., individuals that hatched in the year of capture) were used in this study: the age and sex were determined based on plumage and bill colouration (Robson 2020). Upon capture, birds were ringed with an individually numbered metal ring, and their body mass was recorded (± 0.1 g). Birds were initially housed in groups of four individuals in cages (100 × 30 × 50 cm) placed in an outdoor aviary (3.65 × 3.35 × 2.75 m). However, two cages contained three and two birds, respectively, which was necessary because of the odd number of individuals and avoid animals being kept individually. Food and water were provided ad libitum throughout the study. The birds were fed a mixture of freshly grated carrots, apples, quark, hard-boiled eggs, an insectivorous bird food and ground cat food as a protein supplement, live mealworms daily and occasionally small crickets, grasshoppers and immature Turkestan cockroaches.

After at least ten days of acclimation, the individuals were randomly assigned to receive either IGF-1 or a control treatment. Treatments were started in a staggered manner over two weeks, meaning that the four-day treatment period was started on different calendar days for different birds (the order within each cage was randomised), to minimise the number of experimental birds and thus handling time on any particular day. Controlling for the experimental order in the analyses had no effect on the results. On the morning of the treatment (day 0), we took a baseline blood sample (mean handling time: 190 ± 100 SD sec time measured from entering the aviary) from each target individual and recorded their body mass. Total blood volume was approximately 70 µL, and the average plasma volume collected was 37 µL. Subsequently, we injected subcutaneously 100 µL dispersion containing either slow-release PLGA (poly(lactide-co-glycolide)) microspheres loaded with recombinant human IGF-1 (PeproTech, UK) (treatment; 2.2 mg microspheres containing 272 ng/mg IGF-1) or only the dispersion medium (control). Due to the high structural similarity between human and avian IGF-1, the human peptide has been successfully used in birds (McGuinness and Cogburn 1991; Lodjak et al. 2017; Lendvai et al. 2021).

The PLGA microparticles were designed to release exogenous IGF-1 over several days and have been shown to be a suitable method for this species (Meinel et al. 2001; Luginbuehl et al. 2013; Lendvai et al. 2021; Mahr et al. 2023). To produce the PLGA-microparticles, we conducted microencapsulation of recombinant human IGF-I using a solvent extraction method from a W_1_/O/W_2_ dispersion. The internal aqueous phase (W_1_), comprising IGF-1, 10 mmol/L sodium succinate, 140 mmol/L sodium chloride (pH 6.0), and bovine serum albumin as a stabiliser, was emulsified with a solution of PLGA in dichloromethane (O) through ultrasonication. This W_1_/O dispersion was then introduced into a 5% (w/v) aqueous PVA solution (W_2_) to create, under mechanical stirring, a W_1_/O/W_2_ dispersion. For solvent extraction, the W_1_/O/W_2_ dispersion was diluted with de-ionised water and stirred using a magnetic stirrer. The resulting microspheres were collected on a regenerated cellulose (RC) membrane filter and dried at room temperature overnight under reduced pressure. The microspheres had an IGF-1 loading of 272 ng IGF-I mg/microspheres. Treated birds received a total of 600 ng IGF-1 per injection, with 100 μL of the dispersion administered subcutaneously between the shoulders. The control birds followed the same protocol, except they were only injected with 100 μL of the dispersion medium. The dispersion medium consisted of 1.5% (m/m) carboxymethylcellulose, 5% mannitol and 0.02% polysorbate 80 in sterile saline solution.

Immediately after the treatment, the birds were returned to their cages. Additional blood samples (using the same procedure as above) and body mass measurements were taken after 24 h and 96 h (day 1 and day 4 post-treatment) to assess the short-term physiological effects of the treatment. Once all birds had undergone day 4 post-treatment sampling, birds from half of all cages (chosen randomly) were released back to the aviary, where the cages were placed, while the other half were released into another outdoor aviary (3.7 × 3.5 × 2.2 m). Both aviaries contained dense bundles of reed and cattail, and a water pool of ~ 1 m^2^ surface area to mimic a natural environment. Sufficient branches for perching and small boxes for hiding and resting were provided to enrich the environment. Food (as described above) was provided ad libitum in both aviaries.

At three months post-treatment, between November 20 and 22, 2017, all birds were recaptured to take another blood sample for testing long-term repeatability of circulating IGF-1 levels. Birds were then released back into the aviaries for an additional 13 months (i.e., 16 months in total). Bearded reedlings are short-lived passerines with high juvenile mortality (Peiró 2013). Therefore, the entire study period was sufficiently long to detect enough mortality events for statistical analyses.

Mortality events were recorded on a daily basis. While the immediate cause of mortality remains unknown (autopsy and post-mortem pathology were not performed), most of the birds died in apparently good condition, without any visible injuries, suggesting intrinsic physiological causes of juvenile death. Body mass did not differ between treatment and control groups at any time point (all *p* > 0.2). Birds gained significant amount of mass during acclimation (1.3 g ± 0.22 s.e.m., *p* < 0.001) that remained constant during the short-term phase of the study (day 0–4) and gained additional mass (0.9 g ± 0.26 s.e.m., *p* < 0.001) by November, three months later, indicating good conditions and no adverse effects of captivity in the aviaries. The sexes did not differ in body mass gain at any time point (all *p* > 0.1). After 16 months in captivity, on December 8, 2018, all surviving birds (31%, *n* = 13; 7 controls and 6 treated individuals) were released at the site of capture.

### Physiological measurements

Plasma IGF-1 levels were measured without extraction by an in-house ELISA assay, as described elsewhere (Mahr et al. 2020). MDA was measured by high-performance liquid chromatography, as detailed elsewhere (Vágási et al. 2019).

### Statistical analyses

All statistical analyses were carried out in R version ‘Bird Hippie’ (4.1.2.) (R Core Team 2021). We analysed treatment effects on circulating IGF-1 and MDA levels (both log-transformed) (*n* = 41 individuals) by generalised mixed-effects models (GLMMs) with treatment, sex and sampling time (days 0, 1, and 4) and their interactions as fixed factors, and individual identity as random intercept as implemented in package ‘lme4’ (Bates et al. 2015). To analyse the main effects and interactions, we computed Type III analysis of variance using Satterthwaite’s approximation of degrees of freedom, as implemented in the package ‘lmerTest’ (Kuznetsova et al. 2017). Based on these models, we compared predicted marginal means of the IGF-1 treatment and control groups within each time point and sex and reported these results. These pairwise comparisons were implemented using the function ‘pairs’ in the package ‘emmeans’ (Lenth et al. 2023), and *p*-values were adjusted using the Tukey HSD method. The repeatability of IGF-1 level was estimated using the package ‘rptR’ (Stoffel et al. 2017). Survival analyses were carried out by Aalen’s regression (function ‘aareg’ in package ‘survival’ (Therneau 2009)) that allows for additive effects on the cumulative hazard function. Individuals alive at the end of the study and one individual that escaped from captivity were right-censored in the models. First, we analysed the effect of treatment and sex as factors on the survivorship. Second, we asked how IGF-1 and MDA measured on day 0, 1 and 4 affected survivorship. We included aviary ID in all of the models either as a fixed factor or as ‘strata’ (i.e. calculating a different baseline hazard for each aviary), but this effect never approached statistical significance or altered the conclusions, therefore, it was removed from the models we report here. To avoid overparameterisation of the models, we modelled survival in several steps. First, we considered treatment, sex and their interaction as factors. Next, we included sex and its two-way interactions with both IGF-1 and MDA levels as covariates for the pre-treatment (day 0) and the post-treatment (day 4) periods, respectively. The model structure was then simplified to retain only significant interactions. Finally, based on the earlier models, we constructed a model containing sex and its interaction with either IGF-1 and MDA, found to be influential at the pre-treatment or post-treatment period.

## Results

At the onset of the experiment, neither IGF-1 nor MDA levels were different between the treatment groups (IGF-1: *t* = 0.47, *p* = 0.640; MDA: *t* = 0.88, *p* = 0.382). While pre-treatment MDA levels were higher in males than in females (*t* = 2.03, *p* = 0.049), it was not related to pre-treatment IGF-1 levels (*t* =  – 0.33, *p* = 0.741).

**Fig. 1:**
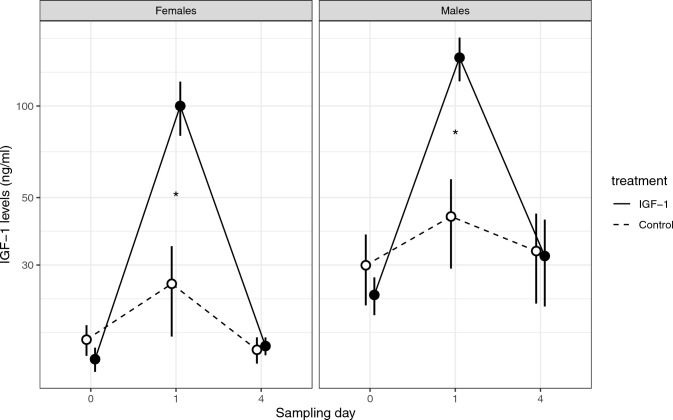
Injection with insulin-like growth factor 1 (IGF-1)-loaded microspheres resulted in a significant increase in circulating IGF-1 levels measured 24 h later (day 1) in captive bearded reedlings, but these effects disappeared by day 4. Mean ± s.e.m. are shown, asterisks denote significant (*p* < 0.05) differences between the treatment and control groups on day 1. The effect of treatment was similar in both sexes, but males had overall higher IGF-1 levels

Hormone treatment increased IGF-1 levels (*F*_2,74_ = 27.13, *p* < 0.001) in both sexes (Fig. 1). Although males had overall higher levels of IGF-1 than females (*F*_1,37_ = 11.25, *p* = 0.002), the magnitude of increase in IGF-1 from day 0 to day 1 was similar in males and females (day × treatment × sex: *F*_2,74_ = 0.02, *p* = 0.978, Fig. 1). IGF-1 levels were similar in the two treatment groups before the manipulation (i.e., day 0) in both sexes (females: *p* = 0.639, males: *p* = 0.674), but it was higher in the treated group than in the control group on day 1 after injection of the IGF-1-loaded microspheres (both sexes: *p* < 0.001, Fig. 1). By day 4, this difference between the two groups disappeared in both sexes (females: *p* = 0.835, males: *p* = 0.948, Fig. 1). Individual identity accounted for 17% of variance in IGF-1 not attributable to fixed effects (conditional *R*^2^ = 0.66). Inter-individual variation in IGF-1 levels remained consistent throughout the study period, resulting in significant repeatability over three months (controlling for day, sex and treatment: *R* = 0.34 (± 0.16 SE), 95% confidence interval: 0.06–0.66, *p* = 0.022, *n* = 29).

**Fig. 2:**
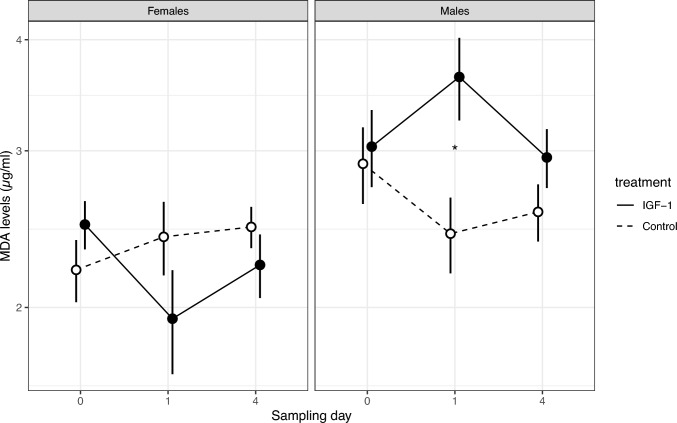
Injection with insulin-like growth factor 1 (IGF-1)-loaded microspheres resulted in a significant increase in cellular oxidative damage (malondialdehyde, MDA) measured 24 h later (day 1) in male, but not in female bearded reedlings. Mean ± s.e.m. are shown, asterisk denotes significant (*p* < 0.05) difference between the treatment and control groups in males on day 1. The effect of treatment was sex-dependent and males had overall higher MDA levels

The hormone treatment induced a significant difference between the treatment groups in MDA levels in a sex-specific manner, indicating that the IGF-1 treatment had opposing effects on MDA levels in males and females (day × treatment × sex: *F*_2,72_ = 3.42, *p* = 0.038, Fig. 2). Post-hoc analyses revealed that before the treatment (day 0), there was no difference between experimental groups in their MDA levels in either sex (females: *p* = 0.382, males: *p* = 0.804). However, IGF-1-injected males had higher MDA on day 1 than control males (*p* = 0.002), but this difference disappeared by day 4 (*p* = 0.316, Fig. 2). In contrast, while females appeared to show the opposite pattern, the difference in MDA between the hormone-treated and control groups did not reach statistical significance on either day 1 or day 4 (*p* = 0.172 and 0.493, respectively). Individual variation explained 7.1% of variance in MDA not attributable to fixed effects (conditional *R*^2^ = 0.26).

**Fig. 3:**
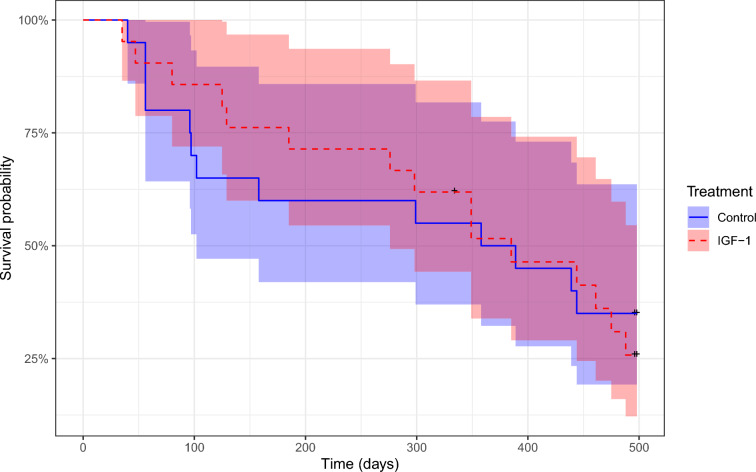
Insulin-like growth factor 1 (IGF-1) treatment did not affect survivorship in bearded reedlings. The solid and the dashed lines represent the Kaplan–Meier survival curves for the IGF-1 treatment and control birds, respectively, and shaded areas denote the corresponding 95% confidence intervals. Cross symbols show censored values. Treatment groups did not differ in survivorship

Survivorship over 16 months did not differ between the IGF-1-treated and control groups (*z* = 0.28, *p* = 0.773, Fig. 3) or between sexes (*z* = 0.15, *p* = 0.883) and the interaction of these two predictors was also non-significant (*z* = 0.50, *p* = 0.620). Birds with higher pre-treatment (day 0) IGF-1 levels were slightly more likely to survive (*z* =  – 2.65, *p* = 0.008), while pre-treatment MDA and sex had no significant effect in the model (MDA: *z* = 0.26, *p* = 0.799, sex: *z* = 0.42, *p* = 0.675). Neither peak (day 1) MDA and IGF-1 levels nor sex affected survivorship (all *p* > 0.610). However, MDA on day 4 was associated with survivorship in a sex-specific manner: relatively higher MDA levels on day 4 co-occurred with higher mortality in males (*z* = 2.07, *p* = 0.039), while females showed the opposite pattern (*z* =   –  2.09, *p* = 0.037). IGF-1 on day 4 showed no such relationship with survival and remained non-significant in both sexes (females: *z* =   –  0.37, *p* = 0.711, males: *z* = 0.34, *p* = 0.737). Finally, we combined the significant effects of pre-treatment IGF-1 and post-peak MDA levels in a sex-specific model, corroborating the conclusions for MDA, but the effect of pre-treatment IGF-1 was only significant in males (Table 1).

**Table 1:** Survival model predicts that the likelihood of mortality increases over time, but higher pre-treatment (day 0) insulin-like growth factor 1 (IGF-1) levels reduce mortality in males

Fixed effects	Estimate ± s.e.m	*z*	*p*
Baseline hazard	0.187 ± 0.081	2.30	0.022
Sex (males)	–0.155 ± 0.085	–1.81	0.070
Pre-treatment IGF-1 (day 0)	0.002 ± 0.001	1.64	0.100
Pre-treatment IGF-1 × sex (males)	–0.004 ± 0.002	–2.46	0.014
Post-peak MDA (day 4)	–0.075 ± 0.032	–2.36	0.019
Post-peak MDA × sex (males)	0.090 ± 0.034	2.64	0.008

Post-peak (day 4) levels of malondialdehyde (MDA), a marker of oxidative damage to lipids, increase mortality in males, while it has the opposite effect in females

## Discussion

IGF-1 is a pleiotropic hormone that has antagonistic effects on life-history traits (Dantzer and Swanson 2012; Lodjak and Verhulst 2020), but the adaptive value of among-individual variation in its plasma levels remains unknown. Higher IGF-1 titres might be associated with increased mortality in reptiles and mammals, though effect sizes differ between studies and according to the sex and age of individuals (Holzenberger et al. 2003; Andreassen et al. 2009; Sparkman et al. 2009; Milman et al. 2016; Garratt et al. 2017; Lewin et al. 2017). Phylogenetic comparative analyses also report a negative relationship between circulating IGF-1 levels and lifespan in birds and mammals (Swanson and Dantzer 2014; Lodjak et al. 2018). Although the exact mechanism of such increased mortality remains uncertain, several studies suggested oxidative stress as a mediatory agent (Holzenberger et al. 2003; Tatar et al. 2003; Brys et al. 2007; Kenyon 2010; Dantzer and Swanson 2012).

Here, for the first time, we showed experimental support for the hypothesis that an elevation of circulating IGF-1 levels may cause oxidative damage at short-term in birds originating from a wild population. This result is consistent with a previous correlational study where circulating baseline levels of IGF-1 were positively associated with MDA in adult house sparrows (*Passer domesticus*) (Vágási et al. 2020). However, the role of IGF-1 in oxidative stress is complex. While higher activity of the IIS pathway is associated with oxidative damage, concurrently, it also upregulates antioxidant defences. For example, a study on nestling pied flycatchers (*Ficedula hypoleuca*) found that daily IGF-1 injections increased the levels of the antioxidant enzyme glutathione peroxidase (Lodjak and Mägi 2017), which might reflect lowered oxidative stress and/or up-regulated antioxidant activity in response to oxidative stress. This upregulation of antioxidant defences may contribute to the protective effects of IGF-1 in specific tissues (especially in the neural system), so much so that in clinical settings, the therapeutic use of IGF-1 is also tested to prevent neurodegenerative disorders (Ayadi et al. 2016; Arjunan et al. 2023). However, enhanced activity of the IGF-1 system is known to generate reactive oxygen species and may lead to lipid peroxidation in rodents (Papaconstantinou 2009; Elis et al. 2011) and systemic augmentation of IGF-1 is associated with an increase of all-cause mortality in humans (Andreassen et al. 2009).

This paradoxical position of IGF-1 in oxidative balance regulation may be partly due to sexual differences in its effect (May 2007; Elis et al. 2011). In this context, we demonstrate that the IGF-1-induced oxidative damage showed marked sexual differences: while the treatment equally increased IGF-1 in males and females, it induced transient oxidative damage in males only, while in females, there was no difference between the treatment groups and they tended to show the opposite pattern. Experimentally-induced IGF-1 levels remained in the natural physiological range of this hormone in this species, as in previous studies, we found that some individuals had similarly high or even higher IGF-1 values than the day 1 experimental birds in this study (Mahr et al. 2020, 2023).

As IGF-1 concentration returned to pre-treatment levels at day 4, the difference in oxidative damage also disappeared between the groups. Microspheres were found to release encapsulated IGF-1 over several days in mice (e.g. Luginbuehl et al. 2013), and in a follow-up study in the bearded reedlings, we also found significant elevation of IGF-1 levels for up to 3 days post-injection. However, after temporary regression, another wave of release sustained elevated levels up to 7 days following the injection of IGF-1 loaded microspheres (Mahr et al. 2023). Whereas treatment effects disappeared by day 4 in the current study, it is remarkable that a single injection with microspheres achieved a sustained increase in IGF-1 for at least 24 h (and potentially more), which is considerably longer than the average half-life (32 min, regardless of the dose) of simple IGF-1 injections used in previous studies (McGuinness and Cogburn 1991).

Although MDA levels also returned to baseline by day 4, males that persistently had relatively higher post-peak oxidative damage levels were more likely to die. Intriguingly, females showed the opposite pattern, where relatively lower MDA levels were associated with higher mortality. Males had overall higher IGF-1 and MDA levels than females and were more susceptible to IGF-1-induced oxidative damage. Studies in mice and humans found the opposite pattern, where females seem more sensitive to variation in IGF-1 levels (Holzenberger et al. 2003; Van Heemst et al. 2005; Elis et al. 2011; Xu et al. 2014). This is remarkable because compared with mammals, in birds, males tend to have longer lifespans (Bronikowski et al. 2022). Hence, our study indicates that the IGF-1- related physiology and oxidative damage may contribute to sex-specific mortality patterns.

Notably, higher baseline IGF-1 (but not MDA) levels measured before the treatment were associated with lower mortality (especially in males). This result was unexpected since higher IGF-1 activity has been linked to higher mortality in various species (see above). However, it is also noteworthy that individuals exhibit large, repeatable natural variation in IGF-1 levels, which may be the result of individual optimisation (recently coined as the Optimal Endocrine Phenotype Hypothesis) (Bonier and Cox 2020). In this context, most of the inter-individual variation of IGF-1 levels may reflect adaptive plastic responses to variation in environmental or internal conditions, where individuals express endocrine phenotypes that are optimal in their current conditions but which differ among them. Therefore, high-quality individuals (e.g., good health or nutritional status) may afford to bear the costs of elevated IGF-1 levels (e.g. in terms of oxidative damage, accelerated ageing or increased risk of cancer (Shanmugalingam et al. 2016; Montoya et al. 2022b; Nelson et al. 2023)) while benefiting from its fitness-enhancing effects (e.g. boosting fecundity or anti-inflammatory responses) (Higashi et al. 2010) as expected for wild species exposed to forces of natural selection. Therefore, despite the high levels of circulating IGF-1, the overall balance of its antagonistic effects may still be positive for high-quality individuals, who, by definition, have better survival prospects. However, it is crucial to recognise that the association between natural variations in pre-treatment IGF-1 or post-treatment MDA levels and observed mortality patterns is correlational, and a direct causal relationship cannot be concluded. Birds with diverse IGF-1 or MDA levels likely exhibit variations in numerous physiological aspects. For instance, a recent study on male Japanese quails (*Coturnix japonica*) reported a positive link between IGF-1 and immune response while finding no relationship between MDA and IGF-1 (Montoya et al. 2023). IGF-1 levels may also reflect short-term nutritional status (Lodjak et al. 2023). We do not have information about the immune or nutritional status of the birds in the aviary. Furthermore, the intricacies of IGF-1 signalling involve complex processes, including interactions with various binding globulins that can alter hormone signalling (McMurtry et al. 1997; Reindl and Sheridan 2012; Allard and Duan 2018). Tissue-specific modulation of receptor densities or local IGF-1 production acting in autocrine or paracrine manners also add layers of complexity to regulation. Additionally, given IGF-1’s ability to bind to insulin receptors, potential alterations in glucose metabolism may also influence avian longevity (Montoya et al. 2018, 2022a). These intricate mechanisms could contribute to or obscure any relationship between baseline IGF-1 or MDA levels and mortality.

We measured survival in a semi-natural environment under ad libitum diet regime and shelter from predators but under exposure to inclement weather, parasites and other pathogens. Fluctuations in environmental conditions and stress stimuli is known to substantially reorganise the physiological network and, therefore, alter the adaptive value of a given endocrine phenotype (Vágási et al. 2020). IGF-1 levels showed high inter-individual variability and significant repeatability over three months, indicating that the circulating levels of this hormone may be a consistent individual phenotypic marker, subject to individual optimisation. Whether individuals with naturally high IGF-1 levels also realise fitness advantages under more challenging natural conditions remains to be investigated.

## Data Availability

All data supporting the results is available from the corresponding author (ÁZL) upon request.

## References

[CR1] Aksu I, Baykara B, Kiray M et al (2013) Serum IGF-1 levels correlate negatively to liver damage in diabetic rats. Biotech Histochem 88:194–201. 10.3109/10520295.2012.758311 23331186 10.3109/10520295.2012.758311

[CR2] Allard JB, Duan C (2018) IGF-binding proteins: why do they exist and why are there so many? Front Endocrinol 9:117 10.3389/fendo.2018.00117 PMC5900387 29686648

[CR3] Andreassen M, Raymond I, Kistorp C et al (2009) IGF1 as predictor of all cause mortality and cardiovascular disease in an elderly population. Eur J Endocrinol 160:25–31. 10.1530/EJE-08-0452 18931092 10.1530/EJE-08-0452

[CR4] Arjunan A, Sah DK, Woo M, Song J (2023) Identification of the molecular mechanism of insulin-like growth factor-1 (IGF-1): a promising therapeutic target for neurodegenerative diseases associated with metabolic syndrome. Cell Biosci 13:16. 10.1186/s13578-023-00966-z 36691085 10.1186/s13578-023-00966-z PMC9872444

[CR5] Ayadi AE, Zigmond MJ, Smith AD (2016) IGF-1 protects dopamine neurons against oxidative stress: association with changes in phosphokinases. Exp Brain Res 234:1863–1873. 10.1007/s00221-016-4572-1 26894890 10.1007/s00221-016-4572-1 PMC4893922

[CR6] Bartke A (2017) Somatic growth, aging, and longevity. NPJ Aging Mech Dis 3:14. 10.1038/s41514-017-0014-y 28970944 10.1038/s41514-017-0014-y PMC5622030

[CR7] Bates D, Mächler M, Bolker B, Walker S (2015) Fitting linear mixed-effects models using lme4. J Stat Softw 67:1–48. 10.18637/jss.v067.i01

[CR8] Bonier F, Cox RM (2020) Do hormone manipulations reduce fitness? A meta-analytic test of the optimal endocrine phenotype hypothesis. Mol Cell Endocrinol 500:110640. 10.1016/j.mce.2019.110640 31715223 10.1016/j.mce.2019.110640

[CR9] Bronikowski AM, Meisel RP, Biga PR et al (2022) Sex-specific aging in animals: perspective and future directions. Aging Cell 21:e13542. 10.1111/acel.13542 35072344 10.1111/acel.13542 PMC8844111

[CR10] Brys K, Vanfleteren JR, Braeckman BP (2007) Testing the rate-of-living/oxidative damage theory of aging in the nematode model *Caenorhabditis elegans*. Exp Gerontol 42:845–851. 10.1016/j.exger.2007.02.004 17379464 10.1016/j.exger.2007.02.004

[CR11] Dantzer B, Swanson EM (2012) Mediation of vertebrate life histories via insulin-like growth factor-1. Biol Rev 87:414–429. 10.1111/j.1469-185X.2011.00204.x 21981025 10.1111/j.1469-185X.2011.00204.x

[CR12] Del Rio D, Stewart AJ, Pellegrini N (2005) A review of recent studies on malondialdehyde as toxic molecule and biological marker of oxidative stress. Nutr Metab Cardiovasc Dis 15:316–328. 10.1016/j.numecd.2005.05.003 16054557 10.1016/j.numecd.2005.05.003

[CR13] Elis S, Wu Y, Courtland H-W et al (2011) Increased serum IGF-1 levels protect the musculoskeletal system but are associated with elevated oxidative stress markers and increased mortality independent of tissue igf1 gene expression. Aging Cell 10:547–550. 10.1111/j.1474-9726.2011.00683.x 21418509 10.1111/j.1474-9726.2011.00683.x PMC3094487

[CR14] Garratt M, Nakagawa S, Simons MJP (2017) Life-span extension with reduced somatotrophic signaling: moderation of aging effect by signal type, sex, and experimental cohort. J Gerontol A Biol Sci Med Sci 72:1620–1626. 10.1093/gerona/glx010 28207064 10.1093/gerona/glx010 PMC5861954

[CR15] Higashi Y, Sukhanov S, Anwar A et al (2010) IGF-1, oxidative stress and atheroprotection. Trends Endocrinol Metab 21:245–254. 10.1016/j.tem.2009.12.005 20071192 10.1016/j.tem.2009.12.005 PMC2848911

[CR16] Hoi H, Griggio M (2012) Bearded reedlings adjust their pair-bond behaviour in relation to the sex and attractiveness of unpaired conspecifics. PLoS One 7:e32806. 10.1371/journal.pone.0032806 22393449 10.1371/journal.pone.0032806 PMC3290599

[CR17] Holzenberger M, Dupont J, Ducos B et al (2003) IGF-1 receptor regulates lifespan and resistance to oxidative stress in mice. Nature 421:182–187. 10.1038/nature01298 12483226 10.1038/nature01298

[CR18] Kenyon CJ (2010) The genetics of ageing. Nature 464:504–512. 10.1038/nature08980 20336132 10.1038/nature08980

[CR19] Kuznetsova A, Brockhoff PB, Christensen RHB (2017) lmerTest package: tests in linear mixed effects models. J Stat Softw 82:1–26. 10.18637/jss.v082.i13

[CR20] Lendvai ÁZ (2023) Bearded reedling (*Panurus biarmicus*): the biology of a remarkable bird – a review of the recent literature. Ornis Hung 31:1–1. 10.2478/orhu-2023-0001 10.2478/orhu-2023-0001

[CR21] Lendvai ÁZ, Tóth Z, Mahr K et al (2021) Effects of experimental increase in insulin-like growth factor 1 on feather growth rate, moult intensity and feather quality in a passerine bird. J Exp Biol. 10.1242/jeb.242481 34124749 10.1242/jeb.242481

[CR22] Lenth RV, Bolker B, Buerkner P et al (2023) emmeans: estimated marginal means, aka least-squares means. R package version 1.9.0. https://CRAN.R-project.org/package=emmeans

[CR23] Lewin N, Swanson EM, Williams BL, Holekamp KE (2017) Juvenile concentrations of IGF-1 predict life-history trade-offs in a wild mammal. Funct Ecol 31:894–902. 10.1111/1365-2435.12808 10.1111/1365-2435.12808

[CR24] Lind MI, Ravindran S, Sekajova Z et al (2019) Experimentally reduced insulin/IGF-1 signaling in adulthood extends lifespan of parents and improves Darwinian fitness of their offspring. Evol Lett 3:207–216. 10.1002/evl3.108 31007945 10.1002/evl3.108 PMC6457396

[CR25] Lodjak J, Mägi M (2017) Crosstalk between growth and somatic maintenance in young animals. J Avian Biol 48:1360–1363. 10.1111/jav.01519 10.1111/jav.01519

[CR26] Lodjak J, Verhulst S (2020) Insulin-like growth factor 1 of wild vertebrates in a life-history context. Mol Cell Endocrinol 518:110978. 10.1016/j.mce.2020.110978 32798584 10.1016/j.mce.2020.110978

[CR27] Lodjak J, Mägi M, Sild E, Mänd R (2017) Causal link between insulin-like growth factor 1 and growth in nestlings of a wild passerine bird. Funct Ecol 31:184–191. 10.1111/1365-2435.12679 10.1111/1365-2435.12679

[CR28] Lodjak J, Mänd R, Mägi M (2018) Insulin-like growth factor 1 and life-history evolution of passerine birds. Funct Ecol 32:313–323. 10.1111/1365-2435.12993 10.1111/1365-2435.12993

[CR29] Lodjak J, Boonekamp J, Lendvai ÁZ, Verhulst S (2023) Short- and long-term effects of nutritional state on IGF-1 levels in nestlings of a wild passerine. Oecologia. 10.1007/s00442-023-05445-3 37676486 10.1007/s00442-023-05445-3 PMC10615909

[CR30] Luginbuehl V, Zoidis E, Meinel L et al (2013) Impact of IGF-I release kinetics on bone healing: a preliminary study in sheep. Eur J Pharm Biopharm 85:99–106. 10.1016/j.ejpb.2013.03.004 23958321 10.1016/j.ejpb.2013.03.004

[CR31] Mahr K, Vincze O, Tóth Z et al (2020) Insulin-like growth factor 1 is related to the expression of plumage traits in a passerine species. Behav Ecol Sociobiol 74:39. 10.1007/s00265-020-2821-6 10.1007/s00265-020-2821-6

[CR32] Mahr K, Anzengruber M, Hellerschmid A et al (2023) Biocompatible polymeric microparticles serve as novel and reliable vehicles for exogenous hormone manipulations in passerines. Gen Comp Endocrinol 336:114234. 10.1016/j.ygcen.2023.114234 36791824 10.1016/j.ygcen.2023.114234

[CR33] May RC (2007) Gender, immunity and the regulation of longevity. BioEssays 29:795–802. 10.1002/bies.20614 17621669 10.1002/bies.20614

[CR34] McGuinness MC, Cogburn LA (1991) Response of young broiler chickens to chronic injection of recombinant-derived human insulin-like growth factor-I. Domest Anim Endocrinol 8:611–620. 10.1016/0739-7240(91)90031-E 1786708 10.1016/0739-7240(91)90031-E

[CR35] McMurtry JP, Francis GL, Upton Z (1997) Insulin-like growth factors in poultry. Domest Anim Endocrinol 14:199–229. 10.1016/S0739-7240(97)00019-2 9260060 10.1016/S0739-7240(97)00019-2

[CR36] Meinel L, Illi OE, Zapf J et al (2001) Stabilizing insulin-like growth factor-I in poly (D, L-lactide-co-glycolide) microspheres. J Controlled Release 70:193–202 10.1016/S0168-3659(00)00352-7 11166419

[CR37] Milman S, Huffman DM, Barzilai N (2016) The somatotropic axis in human aging: framework for the current state of knowledge and future research. Cell Metab 23:980–989. 10.1016/j.cmet.2016.05.014 27304500 10.1016/j.cmet.2016.05.014 PMC4919980

[CR38] Monaghan P, Metcalfe NB, Torres R (2009) Oxidative stress as a mediator of life history trade-offs: mechanisms, measurements and interpretation. Ecol Lett 12:75–92. 10.1111/j.1461-0248.2008.01258.x 19016828 10.1111/j.1461-0248.2008.01258.x

[CR39] Montivero AJ, Ghersi MS, Silvero CMJ et al (2021) Early IGF-1 gene therapy prevented oxidative stress and cognitive deficits induced by traumatic brain injury. Front Pharmacol 12:672392 34234671 10.3389/fphar.2021.672392 PMC8255687

[CR40] Montoya B, Briga M, Jimeno B et al (2018) Baseline glucose level is an individual trait that is negatively associated with lifespan and increases due to adverse environmental conditions during development and adulthood. J Comp Physiol B 188:517–526. 10.1007/s00360-017-1143-0 29313093 10.1007/s00360-017-1143-0

[CR41] Montoya B, Torres R, Hernández A, Alejandro V (2023) IGF-1 levels increase during an immune but not an oxidative challenge in an avian model, the Japanese quail. Physiol Biochem Zool. 10.1086/728771 38237191 10.1086/728771

[CR42] Montoya B, Briga M, Jimeno B, Verhulst S (2022a) Glucose tolerance predicts survival in old zebra finches. J Exp Biol. 10.1242/jeb.243205 35574668 10.1242/jeb.243205

[CR43] Montoya B, Tóth Z, Lendvai ÁZ et al (2022b) Does IGF-1 shape life-history trade-offs? Opposite associations of IGF-1 with telomere length and body size in a free-living bird. Front Ecol Evol 10:853674 10.3389/fevo.2022.853674

[CR44] Nelson VK, Nuli MV, Mastanaiah J et al (2023) Reactive oxygen species mediated apoptotic death of colon cancer cells: therapeutic potential of plant derived alkaloids. Front Endocrinol 14:1201198. 10.3389/fendo.2023.1201198 10.3389/fendo.2023.1201198 PMC10408138 37560308

[CR45] Pamplona R, Barja G (2011) An evolutionary comparative scan for longevity-related oxidative stress resistance mechanisms in homeotherms. Biogerontology 12:409–435. 10.1007/s10522-011-9348-1 21755337 10.1007/s10522-011-9348-1

[CR46] Papaconstantinou J (2009) Insulin/IGF-1 and ROS signaling pathway cross-talk in aging and longevity determination. Mol Cell Endocrinol 299:89–100. 10.1016/j.mce.2008.11.025 19103250 10.1016/j.mce.2008.11.025 PMC2873688

[CR47] Peiró IG (2013) Movements, sex-ratios, recovery rates and longevity of the bearded reedling *Panurus biarmicus* in Iberia. Ringing Migr 28:50–52. 10.1080/03078698.2013.810855 10.1080/03078698.2013.810855

[CR48] Pinto-Benito D, Paradela-Leal C, Ganchala D et al (2022) IGF-1 regulates astrocytic phagocytosis and inflammation through the p110α isoform of PI3K in a sex-specific manner. Glia 70:1153–1169. 10.1002/glia.24163 35175663 10.1002/glia.24163 PMC9305764

[CR49] R Core Team (2021) R: A language and environment for statistical computing. R Foundation for Statistical Computing, URL http://www.R-project.org, Vienna, Austria

[CR50] Reindl KM, Sheridan MA (2012) Peripheral regulation of the growth hormone-insulin-like growth factor system in fish and other vertebrates. Comp Biochem Physiol A Mol Integr Physiol 163:231–245. 10.1016/j.cbpa.2012.08.003 22909791 10.1016/j.cbpa.2012.08.003

[CR51] Robson C (2020) Bearded reedling - *Panurus biarmicus* - Birds of the World. In: Birds of the World (del Hoyo J, Elliott A, Sargatal J, Christie DA, and de Juana E, Editors). Cornell Lab of Ornithology, Ithaca

[CR52] Romero-Pujante M, Hoi H, Blomqvist D, Valera F (2002) Tail length and mutual mate choice in Bearded Tits (*Panurus biarmicus*). Ethology 108:885–895. 10.1046/j.1439-0310.2002.00821.x 10.1046/j.1439-0310.2002.00821.x

[CR53] Shanmugalingam T, Bosco C, Ridley AJ, Van Hemelrijck M (2016) Is there a role for IGF-1 in the development of second primary cancers? Cancer Med 5:3353–3367. 10.1002/cam4.871 27734632 10.1002/cam4.871 PMC5119990

[CR54] Sohal RS, Orr WC (2012) The redox stress hypothesis of aging. Free Radic Biol Med 52:539–555. 10.1016/j.freeradbiomed.2011.10.445 22080087 10.1016/j.freeradbiomed.2011.10.445 PMC3267846

[CR55] Sparkman AM, Vleck CM, Bronikowski AM (2009) Evolutionary ecology of endocrine-mediated life-history variation in the garter snake *Thamnophis elegans*. Ecology 90:720–728. 10.2307/27651035 19341142 10.2307/27651035

[CR56] Stoffel MA, Nakagawa S, Schielzeth H (2017) rptR: repeatability estimation and variance decomposition by generalized linear mixed-effects models. Methods Ecol Evol 8:1639–1644. 10.1111/2041-210X.12797 10.1111/2041-210X.12797

[CR57] Sukhanov S, Higashi Y, Shai S-Y et al (2007) IGF-1 reduces inflammatory responses, suppresses oxidative stress, and decreases atherosclerosis progression in ApoE-deficient mice. Arterioscler Thromb Vasc Biol 27:2684–2690. 10.1161/ATVBAHA.107.156257 17916769 10.1161/ATVBAHA.107.156257

[CR58] Swanson EM, Dantzer B (2014) Insulin-like growth factor-1 is associated with life-history variation across Mammalia. Proc R Soc B Biol Sci 281:20132458. 10.1098/rspb.2013.2458 10.1098/rspb.2013.2458 PMC3973252 24619435

[CR59] Tatar M, Bartke A, Antebi A (2003) The endocrine regulation of aging by insulin-like signals. Science 299:1346–1351. 10.1126/science.1081447 12610294 10.1126/science.1081447

[CR60] Therneau TM (2009) A Package for Survival Analysis in R. R package version 3.5-7. https://CRAN.Rproject.org/package=survival

[CR61] Tóth Z, Ouyang JQ, Lendvai ÁZ (2018) Exploring the mechanistic link between corticosterone and insulin-like growth factor-1 in a wild passerine bird. PeerJ 6:e5936. 10.7717/peerj.5936 30581657 10.7717/peerj.5936 PMC6296332

[CR62] Tóth Z, Mahr K, Ölveczki G et al (2022) Food restriction reveals individual differences in insulin-like growth factor-1 reaction norms. Front Ecol Evol 10:826968 10.3389/fevo.2022.826968

[CR63] Vágási CI, Vincze O, Pătraș L et al (2019) Longevity and life history coevolve with oxidative stress in birds. Funct Ecol 33:152–161. 10.1111/1365-2435.13228 34290466 10.1111/1365-2435.13228 PMC8291348

[CR64] Vágási CI, Tóth Z, Pénzes J et al (2020) The relationship between hormones, glucose, and oxidative damage is condition and stress dependent in a free-living passerine bird. Physiol Biochem Zool 93:466–476. 10.1086/711957 33164671 10.1086/711957 PMC7982133

[CR65] Van Heemst D, Beekman M, Mooijaart SP et al (2005) Reduced insulin/IGF-1 signalling and human longevity. Aging Cell 4:79–85. 10.1111/j.1474-9728.2005.00148.x 15771611 10.1111/j.1474-9728.2005.00148.x

[CR66] Vincze O, Vágási CI, Pénzes J et al (2022) Sexual dimorphism in immune function and oxidative physiology across birds: the role of sexual selection. Ecol Lett 25:958–970. 10.1111/ele.13973 35106902 10.1111/ele.13973 PMC9305230

[CR67] Xu J, Gontier G, Chaker Z et al (2014) Longevity effect of IGF-1R+/− mutation depends on genetic background-specific receptor activation. Aging Cell 13:19–28. 10.1111/acel.12145 23898955 10.1111/acel.12145 PMC4326867

